# Preclinical characterization and *in silico* safety assessment of three virulent bacteriophages targeting carbapenem-resistant uropathogenic *Escherichia coli*

**DOI:** 10.1007/s10123-024-00508-8

**Published:** 2024-03-22

**Authors:** Gunaraj Dhungana, Roshan Nepal, Ghais Houtak, George Bouras, Sarah Vreugde, Rajani Malla

**Affiliations:** 1 Central Department of Biotechnology, Institute of Science and Technology, Tribhuvan University, Kirtipur, Nepal; 2 Government of Nepal, Nepal Health Research Council, Kathmandu, Nepal; 3 Adelaide Medical School, Faculty of Health and Medical Sciences, The University of Adelaide, Adelaide, SA Australia; 4 The Department of Surgery-Otolaryngology Head and Neck Surgery, The Basil Hetzel Institute for Translational Health Research, Central Adelaide Local Health Network, Adelaide, South Australia Australia

**Keywords:** Phage, Phage therapy, Lytic phage, Virulent phage, Carbapenem-resistant *Enterobacteriaceae*, Antimicrobial resistance, AMR, Alternative medicine

## Abstract

Phage therapy has recently been revitalized in the West with many successful applications against multi-drug-resistant bacterial infections. However, the lack of geographically diverse bacteriophage (phage) genomes has constrained our understanding of phage diversity and its genetics underpinning host specificity, lytic capability, and phage-bacteria co-evolution. This study aims to locally isolate virulent phages against uropathogenic *Escherichia coli* (*E. coli*) and study its phenotypic and genomic features. Three obligately virulent Escherichia phages (øEc_Makalu_001, øEc_Makalu_002, and øEc_Makalu_003) that could infect uropathogenic *E. coli* were isolated and characterized. All three phages belonged to *Krischvirus* genus. One-step growth curve showed that the latent period of the phages ranged from 15 to 20 min, the outbreak period ~ 50 min, and the burst size ranged between 74 and 127 PFU/bacterium. Moreover, the phages could tolerate a pH range of 6 to 9 and a temperature range of 25–37 °C for up to 180 min without significant loss of phage viability. All phages showed a broad host spectrum and could lyse up to 30% of the 35 tested *E. coli* isolate*s*. Genomes of all phages were approximately ~ 163 kb with a gene density of 1.73 gene/kbp and an average gene length of ~ 951 bp. The coding density in all phages was approximately 95%. Putative lysin, holin, endolysin, and spanin genes were found in the genomes of all three phages. All phages were strictly virulent with functional lysis modules and lacked any known virulence or toxin genes and antimicrobial resistance genes. Pre-clinical experimental and genomic analysis suggest these phages may be suitable candidates for therapeutic applications.

**Supplementary Information:**

The online version contains supplementary material available at 10.1007/s10123-024-00508-8.

## Introduction

The World Health Organization (WHO) has identified carbapenem-resistant *Escherichia coli* (*E. coli*) as a critical pathogen, underscoring the need to prioritize research and development of novel antimicrobial agents to combat these pathogens. *E. coli*, a Gram-negative bacterium, is a typical member of the gastrointestinal tract’s normal flora in humans and animals. However, it can act as an opportunistic pathogen, leading to various infections, including intestinal diarrhea, urinary tract infections (UTIs), septicemia, and pneumonia (Murray et al. 2022). As such, understanding the mechanisms of antimicrobial resistance in *E. coli* and developing effective strategies to address its impact on public health is of utmost importance (Kaper et al. 2004). In recent years, treatment of such infections has become complicated due to the resistance towards most available antibiotics, including third- and fourth-generation cephalosporins and even colistin, which is considered a last resort drug (Thaden et al. 2016; Sidjabat and Paterson 2015; Johura et al. 2020). Antibiotic resistance poses significant challenges in developing countries due to lax adherence to treatment protocols, inadequate regulation of prescription drugs, unauthorized access to antibiotics, and excessive usage of antibiotics in livestock for financial gain (Ayukekbong et al. 2017).

Among various alternatives to antibiotics being explored, like the use of antimicrobial peptides (AMP), nanoparticles, and preventive probiotics, bacteriophage therapy (phage therapy) has been gaining renewed attention in the West due to its successful application against antibiotic-resistant bacterial infections (Ghosh et al. 2019; Feizi et al. 2022; Schmidt 2019). Bacteriophage (phage) is a virus that primarily infects bacteria and is assumed to be the most abundant biological entity in the biosphere of the earth (Cobian Guemes et al. 2016). Phages were used to treat bacterial infections before the discovery of antibiotics in the 1940s (Aswani and Shukla 2021). The rigor of phage-based research and its clinical application persisted in Eastern Europe like Poland, the former Soviet Republic of Georgia, and Russia (Wittebole, De Roock and Opal 2014). Phages therapy is effectively used to treat antimicrobial resistance (AMR) bacteria like methicillin-resistant *Staphylococcus aureus* (MRSA) and carbapenem-resistant *Enterobacteriaceae* (Ormälä and Jalasvuori 2013; Xu et al. 2015).

Phages from different natural environments may conserve genetic identity and diversity regardless of their host (Hambly and Suttle 2005). The complete sequence of the phage genome facilitates studies of phage ecology, evolution, biodiversity and genetic novelty (Hatfull and Hendrix 2011). It plays an important role in advancing phage research and foundation of the phage repositories for future use (Cook et al. 2021). Large numbers of sequenced phage genomes have been deposited into public databases; however, some genomic orphans are still yet to be discovered. Furthermore, most of the well-studied phages were isolated and sequenced from the Western world, like the Americas, and Europe, but few phages that infect pathogenic *E. coli* strains from regions outside of Europe and the USA have been well characterized and studied at a molecular level. Moreover, only few phages that can kill pathogenic *E. coli* strains from Southeast Asia have been studied at a molecular level, although the burden of MDR, particularly carbapenem resistance, is high in this region.

This study provides insight into the genetic and biological diversity of *E. coli* phages via morphology and genome-based analysis. The present study provides information on the genome architecture, functional annotation, and sequence homology of three newly isolated phages (from the same environment) to each other and other phage genomes isolated elsewhere. Preclinical studies, including stability assays and sequence analysis, further indicate the phage’s lifestyle and its suitability for therapeutic applications by confirming the absence of virulence genes and AMR genes.

## Materials and methods

### Bacterial strains and media

Three carbapenem-resistant clinical isolates of uropathogenic *E. coli* (M1, M2, and M3) were obtained from the National Public Health Laboratory, Kathmandu (NPHL, Kathmandu), and used as host bacteria for isolation of lytic phages. Upon receiving, the carbapenem resistance was verified by the phenotypic EDTA disc synergy test and molecular confirmation through *bla*_*NDM*_ and *bla*_*KPC*_ gene sequencing (data not shown). Additional uncharacterized uropathogenic clinical isolates (*N* = 50, *E. coli* = *35*, *Klebsiella pneumoniae* = *10, Pseudomonas aeruginosa* = *5*) and a standard laboratory strain (*E. coli* MG1655, from ATCC, Manassas, USA) were recovered from the glycerol stock maintained at Central Department of Biotechnology, Tribhuvan University, Nepal, and used only for host-range analysis. The bacteria were propagated in Luria–Bertani (LB) broth (HiMedia, India) at 37 °C.

### Phage isolation and purification

Phages were isolated from the municipal wastewater canals collected from Kathmandu, Nepal, using a previously described double-layer agar assay (DLAA) (Nepal et al. 2022a). Briefly, the water samples were centrifuged at 3220 × g (Centrifuge 5810 R, Eppendorf, Hamburg, Germany) for 20 min at 4 °C, and the supernatant was filtered through a 0.22-μm pore size Whatman™ syringe filter (Sigma-Aldrich, Missouri, USA). One milliliter of the filtrate was mixed with 100 µl exponentially propagating host bacteria separately (McFarland = 1.0) and left at room temperature without shaking (5 min) for phage adsorption. Three-milliliter semisolid top-agar (tryptic soya agar (TSA), agar = 0.4%, stored at 50 °C, HiMedia, India) was added to the mixture, mixed well by swirling, and poured onto the surface of previously prepared bottom-agar (TSA, agar = 1.0%, HiMedia, India). The plates were left to solidify completely and incubated at 37 °C overnight. The next day, the plates were examined for the presence of plaques. For purification, a single isolated plaque was cut and dissolved in 1.0 mL of sodium chloride-magnesium sulfate (SM) buffer (10 mM Tris–HCl, 10 mM MgSO_4_·7H_2_O, 2% gelatin, and 100 mM NaCl, pH 7.5). The process was repeated three times to obtain a pure phage strain. Further, phages were purified by isopycnic cesium chloride (CsCl) density-gradient ultracentrifugation described previously (Beilstein and Dreiseikelmann 2006; Sambrook and Russell 2001). Briefly, 2.0 mL high titer phage (> 10^12^ PFU/ml) lysate was overlaid onto a three-step CsCl gradient containing 1.5 mL each of 1.6p, 1.5p, and 1.4p density CsCl in an ultracentrifuge tube (Beckman Coulter, California, USA). The tube was centrifuged at 45,000 rpm at 4 °C for 5 h in a Beckman L8-80 M Ultracentrifuge with Ti 70.1 fixed angle rotor (Beckman Coulter, California, USA). The phages were collected from the gray-white band that appeared in the tube and dialyzed overnight in SM buffer using a slide-A-Lyzer dialysis cassette (10,000 MWCO, Thermo Scientific, USA). The phage preparation was finally filtered through a 0.22-μm pore-size Whatman™ syringe filter (Sigma-Aldrich, Missouri, USA). The phage titer was determined by DLAA as mentioned earlier.

### Transmission electron microscopy

The CsCl purified phage lysate was fixed with 2% paraformaldehyde and 2.5% glutaraldehyde. Ten microliter phage lysate was spread on a carbon-coated copper grid and negatively stained with 2% (w/v) uranyl acetate (pH 4.5). The copper grid was dried and examined under the FEI Tecnai T-12 transmission electron microscope (TEM) (FEI Company, Oregon, USA) at an accelerating voltage of 80 kV.

### Host range analysis

Isolated phages were tested for their host range on 50 different multidrug-resistant clinical isolates and a standard laboratory *E. coli* MG1655 strain. Initially, spot assay was done to determine the host range of the phages as described previously (Kutter 2009). Based on the spot assay results, the lysis efficacy of the phages on bacterial strains was assessed by Efficiency of Plating (EoP) with modification. Briefly, 100 µl of an overnight culture of each isolate was mixed individually with 3.0 mL of semi-solid top agar (TSA, agar = 0.4%, temperature = 50 °C) and immediately poured onto bottom agar plates (TSA, agar = 1.0%, previously prepared). The top agar was allowed to solidify at room temperature. Then, 10.0 µl of tenfold serial dilutions of the phage lysate (10^8^ PFU/mL) was spotted on the bacterial lawn and completely absorbed on the top agar. The plates were then incubated overnight at 37 °C. After incubation, the lowest titer of the phage that gave countable plaques was determined and double layer agar assay was done as described above. The number of plaques was counted in each bacterial strain. The EoP was determined by dividing the average number of plaques formed on the tested bacterial strain by the average number of plaques on the original host bacterium. The test was performed in triplicates.

### Temperature and pH stability

The stability of isolated phages at different pH and temperatures was determined according to D’Andrea et al. (2017) with modification. Briefly, known phages lysate (1 × 10^8^ PFU/ml) in SM buffer was adjusted to different pHs ranging from 2 to 12. Phage suspensions were incubated for 60 min at 37 °C and then titrated using a double-layer agar assay as described previously. For temperature stability, known phage lysate (1 × 10^8^ PFU/ml) was aliquoted into the Eppendorf tube and incubated at 25 °C, 37 °C, 50 °C, 60 °C, and 70 °C for up to 180 min and titrated by DLAA. The data were analyzed under an ordinary one-way and two-way analysis of variance (ANOVA).

### One-step growth curve and burst size determination

A one-step growth experiment of the phages was performed as described previously by Heineman and Bull (2007) with slight modifications. Briefly, 500 μl of overnight bacterial culture was added to 9.5 ml of LB broth and incubated at 37 °C (rpm = 200) until culture density reached 10e8 (OD_600_ = 0.5, ~ 1 h). Phage lysate was added (1 × 10^6^, multiplicity of infection [MOI] = 0.001) and incubated at 37 °C for 5 min without shaking. A 100 μl of culture was then transferred to 10 ml of pre-warmed LB (37 °C, 1000 × dilution) for adsorption of phages to the host bacteria. At 5.5 and 6.5 min from the infection, 1 ml culture was transferred to different Eppendorf tubes and 20 μl of supernatant from both tubes was titrated for unabsorbed phage (T1). Then, 50 μl of chloroform was added, vortexed, and 20 μl was titrated again (T2). After this, aliquots of 1 ml were taken at intervals of 5 min for up to 80 min, treated with 50 μl of chloroform and the highest phage titer was noted (T3).

$$\mathrm{Burst\;size}= \frac{\mathrm{highest\;titer\;during\;burst\;}({\text{T}}3)}{\mathrm{initially\;infected\;cells\;}({\text{T}}1-{\text{T}}2)}$$

### Phage DNA extraction, genome analysis and *in silico* safety evaluation

The genomic DNA (gDNA) of the phages øEc_Makalu_001, øEc_Makalu_002, and øEc_Makalu_003 was extracted by using standard phenol–chloroform extraction method as described previously (Malke 1989). The phage gDNA concentrations and the quality check was carried out using a NanoDrop 8000 (NanoDrop Technologies Inc., USA). Whole-genome sequencing was performed by the Illumina Nextseq500 platform (Illumina, USA). The CLC Genomics Workbench 6.0 (Qiagen, USA) at default parameters (minimum contig length, 200; automatic word size, yes; perform scaffolding, yes; mismatch cost, 2; insertion cost, 3; deletion cost, 3; length fraction, 0.5; similarity fraction, 0.8) was used to assemble the genome. Phage genomes were annotated for coding DNA sequences (CDS), tRNA, tmRNA, CRISPRs, virulence factors (VFs), toxins, and antimicrobial resistance genes (ARGs) using Pharokka v1.2.0 (Bouras et al. 2022), with all CDS assigned to a functional category using PHROGs (Terzian et al. 2021) where available. A circular map of the annotated phage genome along with the GC skew (window = 500) and GC content (window = 10) was visualized in R Statistical Software (v4.2.2; R Core Team, 2021) using ‘circlize’ R package (Gu et al. 2014). The lifestyle, order, family, and host of the phages were also computationally predicted through PhageAI (Tynecki et al. 2020).

### Genome comparison and phylogenetic analysis

Basic Local Alignment Search Tool (BLAST) online tool from the NCBI website (https://blast.ncbi.nlm.nih.gov/Blast.cgi) (Altschul et al. 1990) was used for comparative nucleotide homology estimation. The phylogenetic tree was constructed BLASTing the query sequence (Escherichia phage Ec_Makalu_001, NCBI accession = MN894885.1) against the NCBI Viruses database (taxid:10,239) using the ‘fast minimum evolution method’ (max seq difference = 0.75). Only the 50 most common phages were included in the phylogenetic analysis. The tree was further visualized using ‘ggtree’ R package (Yu et al. 2017). Also, the genome-based phylogeny and classification were carried out using a VICTOR web service (https://victor.dsmz.de) (Meier-Kolthoff et al., 2013). All pairwise comparisons of the nucleotide sequences were conducted using the Genome-BLAST Distance Phylogeny (GBDP) method under settings recommended for prokaryotic viruses (Meier-Kolthoff and Göker, 2017).

### Data availability and statistical analysis

The genome sequence data are deposited in NCBI under BioProject accession PRJNA594990 and are publicly available through the following GenBank accession numbers: Escherichia phage Ec_Makalu_001 (MN894885), Escherichia phage Ec_Makalu_002 (MN709127), and Escherichia phage Ec_Makalu_003 (MN882349). All statistical analyses were performed using GraphPad Prism 9 (ver 9.5.) and differences with *p* < 0.05 were considered statistically significant. Additional support data generated in this research and all the raw metadata are available as supplementary data through FigShare: 10.6084/m9.figshare.21739154 (Nepal 2022).

## Results

### Phage isolation and morphological characterization

**Fig. 1:**
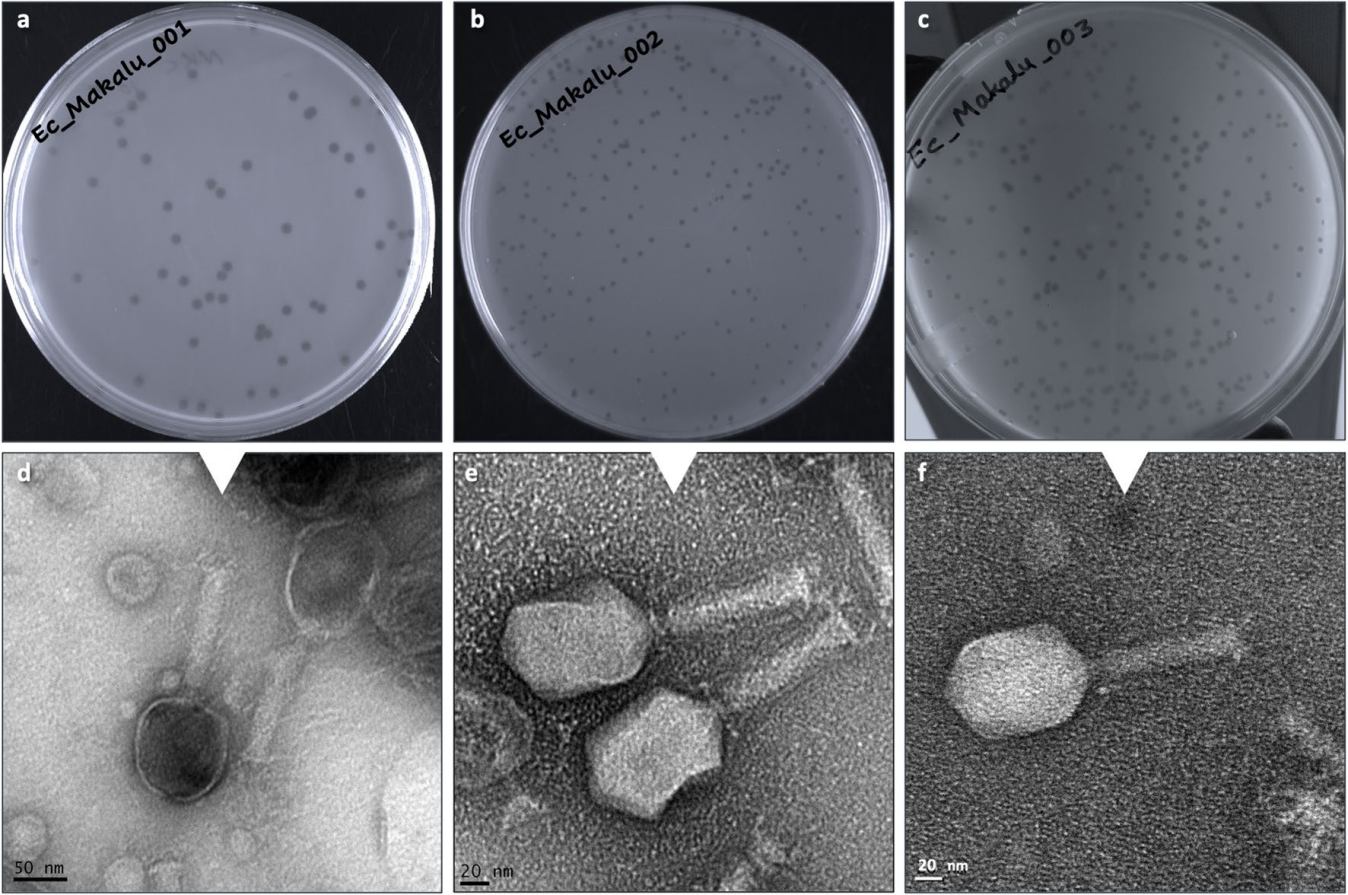
Plaque morphology and transmission electron micrograph of isolated Escherichia phages. Plaques (**a**–**c**) and transmission electron micrograph (**d**–**f**) of Escherichia phage Ec_Makalu_001, Escherichia phage Ec_Makalu_002, and Escherichia phage Ec_Makalu_003 respectively. Panel **b** is already reported in the genome announcement report by the authors and is reproduced here for consistency (Dhungana et al. 2020)

Three phages named as Escherichia phage Ec_Makalu_001, Escherichia phage Ec_Makalu_002, and Escherichia phage Ec_Makalu_003 (hereafter referred to as øEc_Makalu_001, øEc_Makalu_002, øEc_Makalu_003 respectively) were isolated using three different carbapenem-resistant uropathogenic *E. coli* as a host. All phages produced small, clear, non-halo, and round plaques measuring approximately 2–4 mm in diameter on the lawn of the host bacteria (Fig. 1a–c). TEM micrograph of the phage particles revealed that all three phages had icosahedral capsid (head), a long tail with contractile sheath, a baseplate, and tail fibers (Fig. 1d–f). According to latest ICTV guidelines (Virus Taxonomy: 2022 Release) (Turner et al. 2023), all phages belonged to the *Straboviridae* family of *Caudoviricetes* class (Table 1 and 2).

**Table 1:** Morphological classification of phages based on a transmission electron micrograph according to ICTV (the International Committee on Taxonomy of Viruses) guidelines (ICTV 9th report) and latest Virus Taxonomy: 2022 Release (MSL #38)

**Phage name (GenBank)**	**Capsid (in nm**^**a**^**) ± SD**	**Tail (in nm**^**a**^**) ± SD**		**Shape**	**Family**^**b**^ **(Morphotype**^**d**^**)**	**Family**^**c**^
		**Width**	**Length**			
Escherichia phage Ec_Makalu_001	88.9 ± 12.2	19.1 ± 1.0	109 ± 1.0	Icosahedral	*Myoviridae* (A2)	*Straboviridae*
Escherichia phage Ec_Makalu_002	88.5 ± 16.2	18.6 ± 1.4	97.4 ± 1.4	Icosahedral	*Myoviridae* (A2)	*Straboviridae*
Escherichia phage Ec_Makalu_003	83.7 ± 13.0	16.7 ± 1.1	94.3 ± 1.5	Icosahedral	*Myoviridae* (A2)	*Straboviridae*

The head diameter was calculated for isometric capsids. All measurements were made with ImageJ 1.53 k. Three to five individual phage particles were measured for each phage and the standard deviation (SD) was calculated.  * ICTV = The International Committee on Taxonomy of Viruses. ^a^ Nanometre. ^b^ Family is based on ICTV’s 9th report released in 2019 (MSL #35) which is now defunct. We used Myoviridae as family here to be consistent with the already published genome report of Escherichia phage Ec_Makalu_002. ^c^ Family is based on the latest Virus Taxonomy: 2022 release (MSL #38). ^d^ Morphotypes are based on classification by Ackermann (2001)

**Table 2:** Genomic features of three Escherichia phages targeting carbapenem-resistant uropathogenic *Escherichia coli*

**Phage Features**	**Escherichia phage Ec_Makalu_001**	**Escherichia phage Ec_Makalu_002**	**Escherichia phage Ec_Makalu_003**
**NCBI accession**	**MN894885**	**MN709127**	**MN882349**
**Genome features**			
Length (in base pairs)	163,752 bp	164,674 bp	162,966 bp
Guanine-cytosine (G + C) content	40.60%	40.58%	40.68%
Total CDS	284	285	284
Functional genes	114 (40.14%)	114 (40.00%)	113 (39.79%)
Hypothetical genes	170 (59.86%)	171 (60.00%)	171 (60.21%)
Gene density (per kbp)	1.73	1.73	1.74
Average gene size (in bp) Functional genes Hypothetical genes	549 951 279	551 952 283	546 954 276
CDS coverage (coding density)	95.15%	95.33%	95.15%
tRNAs, tmRNAs, and CRISPRs	0	0	0
Integration and excision genes	0	0	0
Virulence factors (VFDB database)	0	0	0
AMR genes (CARD database)	0	0	0
Predicted phage taxonomy* (confidence percentage)			
Realm	*Duplodnaviria*	*Duplodnaviria*	*Duplodnaviria*
Kingdom	*Heunggongvirae*	*Heunggongvirae*	*Heunggongvirae*
Phylum	*Uroviricota*	*Uroviricota*	*Uroviricota*
Class	*Caudoviricetes*	*Caudoviricetes*	*Caudoviricetes*
Family	*Straboviridae*	*Straboviridae*	*Straboviridae*
Genus	*Krischvirus* (99.2%)	*Krischvirus* (99.2%)	*Krischvirus* (99.2%)
Lifestyle	Virulent (96.2%)	Virulent (96.6%)	Virulent (96.7%)

*CDS* coding DNA sequences, *kbp* kilo base pairs, *tRNA* transfer RNA, *tmRNA* transfer-messenger RNA, *CRISPR* clustered regularly interspaced short palindromic repeats, *VFDB* virulence factor database (http://www.mgc.ac.cn/VFs), *CARD* comprehensive antibiotic resistance database (https://card.mcmaster.ca). The genomic features are based on Pharokka output (Bouras et al. 2022); * based on current Virus Taxonomy: 2022 release (MSL #38).

### One-step growth curve and burst size determination

**Fig. 2:**
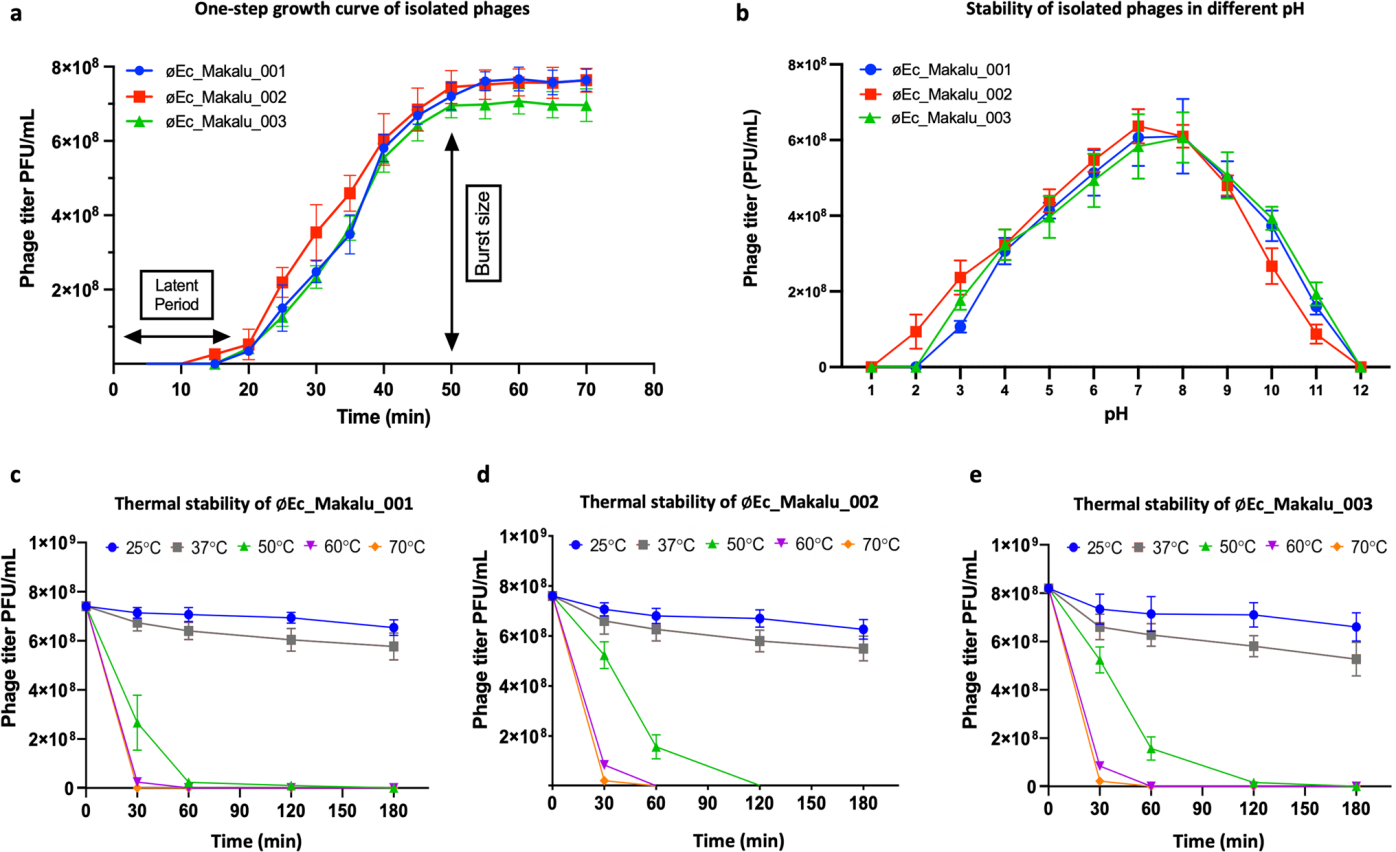
One-step growth curve, pH, and thermal stability of isolated Escherichia phages. **a** One-step growth curve of isolated phages. All phages had their burst around 50 min after infection. **b** pH stability of øEc_Makalu_001, øEc_Makalu_002, and øEc_Makalu_003. Error bars indicate standard deviations of the mean (SEM) of three independent experiments. **c**–**e** Thermal stability of øEc_Makalu_001, øEc_Makalu_002, and øEc_Makalu_003 respectively. All phages are viable at 25 and 37 °C while significantly lose their viability at higher temperatures. Error bars indicate SEM of three independent experiments. The *y*-axis represents PFU/ml

The one-step growth curve experiment was performed to determine the latent period and burst size of all phages. The latent period of 2 phages (øEc_Makalu_001 and øEc_Makalu_003) was 20 min, while øEc_Makalu_002 had a shorter latent period of 15 min (Fig. 2a). The growth of øEc_Makalu_002 and øEc_Makalu_003 reached its plateau at 50 min while the plateau of øEc_Makalu_001 was 55 min (Fig. 2a). The burst size was calculated based on the final titer of the phage and the number of infected bacterial cells. The average burst sizes of øEc_Makalu_001, øEc_Makalu_002, and øEc_Makalu_003 were approximately 127, 74, and 120 phage particles per bacterium respectively (Fig. 2a).

### pH and thermal stability

The stability of isolated phages in different pH was determined by incubating phage lysate in different pH (1–12) for 60 min. The phage viability was significantly unaffected within pH 6 to 9, while viability significantly decreased at pH 3–5 and 10–11. However, phage viability was completely inactivated below pH 2 and above pH 12 (Fig. 2b). Similarly, the thermal stability of isolated phages was determined by incubating the phage lysate at different temperatures (25 °C, 37 °C, 50 °C, 60 °C, and 70 °C) up to 180 min. The titer of all phages did not significantly decrease (*p* > 0.05) at 25 °C and 37 °C for up to 180 min while the phage titer decreased rapidly within 60 min when incubated at 50 °C and lost their viability completely after 180 min (Fig. 2c–e). The viability of phage decreased significantly at or above 60 °C within 30 min.

### Host spectrum

**Fig. 3:**
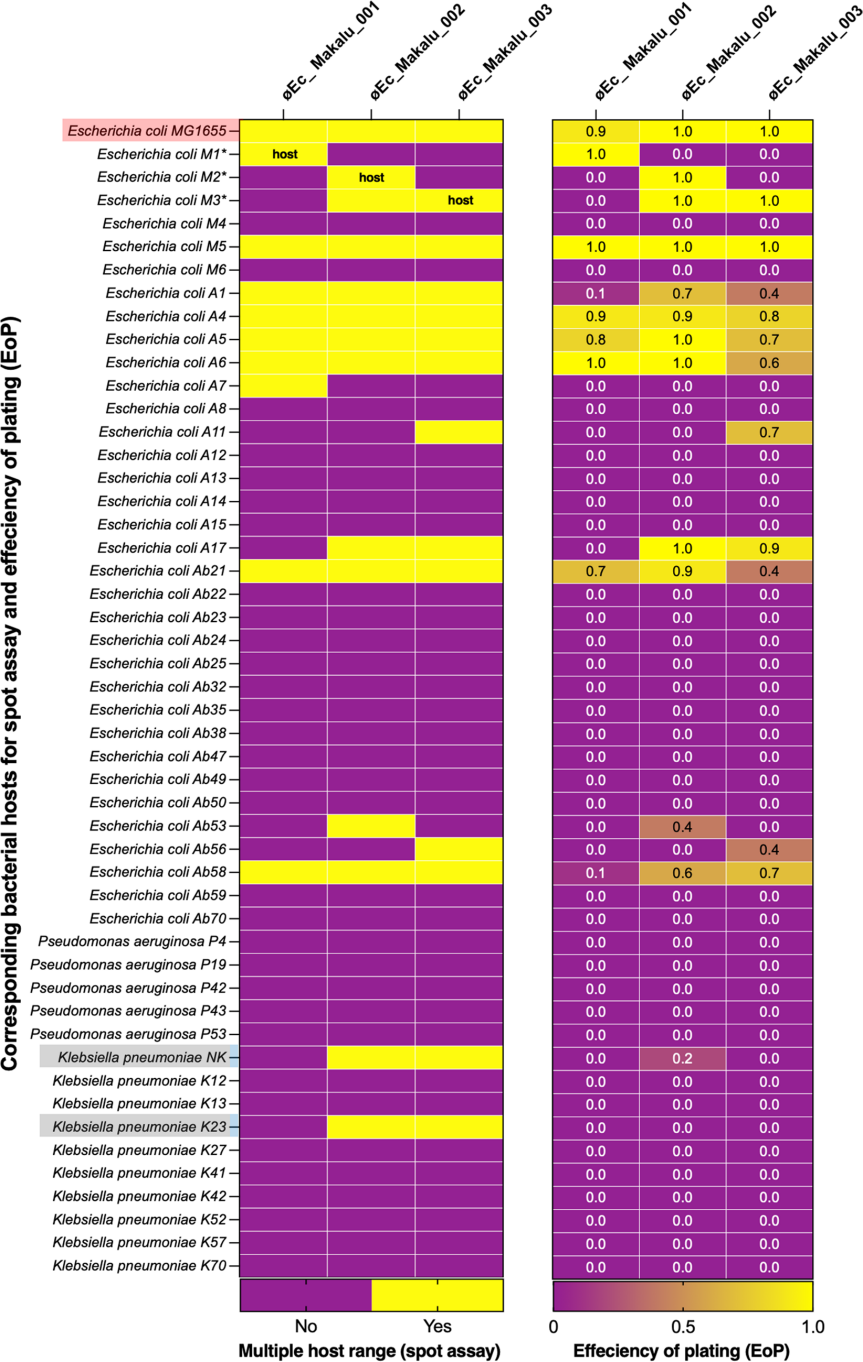
Multiple host range spectrum of isolated Escherichia phages by spot assay and efficiency of plating (EoP). øEc_Makalu_001 lysed 28.5% (10/35) *E. coli* strains only, while øEc_Makalu_002 and øEc_Makalu_003 could kill 34.2% (12/35) *E. coli* strains as well as 20% (2/10) *K. pneumoniae* stains. All three phages did not show any lytic activity on *P. aeruginosa* strains. The EoP analysis revealed that the phages had low to high lysis ability (EoP = 0.1 to 1) among all the spot test-positive *E. coli* strains. Phages øEc_Makalu_002 and øEc_Makalu_003 also showed inter-genus lysis activity in two of the *K. pneumoniae* isolates but the plating efficiency was low (EoP = 0.0 to 0.2)

Host range spectrum of øEc_Makalu_001, øEc_Makalu_002, and øEc_Makalu_003 was evaluated using 50 different uropathogenic clinical isolates (35 *E. coli*, 10 *K. pneumoniae*, and 5 *P. aeruginosa*). Among the 3 phages, øEc_Makalu_001 lysed 28.5% (10/35) *E. coli* strains, while øEc_Makalu_002 and øEc_Makalu_003 could lyse 34.2% (12/35) *E. coli* strains as well as 20% (2/10) of the *K. pneumoniae* stains. No lytic activity against *P. aeruginosa* was observed among the three phages (Fig. 3). Further, EoP was performed to evaluate the ability of phages to produce plaque in bacterial strains other than their primary host. The EoP analysis revealed that the phages had low to high lysis ability (EoP range = 0.1 to 1) among all the spot test-positive *E. coli* strains. Phages øEc_Makalu_002 and øEc_Makalu_003 also showed inter-genus lysis activity in two of the *K. pneumoniae* isolates, but the plating efficiency was low (EoP = 0.0 to 0.2, Fig. 3). All three phages produced plaques on the laboratory strain of *E. coli* MG1655 (EoP = 0.9 to 1.0).

### Genomic features

The genomes of øEc_Makalu_001, øEc_Makalu_002, and øEc_Makalu_003 were composed of a linear double-stranded DNA of 163,752 bp (284 genes), 164,674 bp (285 genes), and 162,966 (284 genes) bp in length respectively. The GC content (~ 40.6%) and gene density (1.7 genes/kilo-base pairs) of all three phages were lower compared to its bacterial host *E. coli* (GC =  ~ 50.6%, gene density = 0.5–1.0 gene/kilo-base pairs). The CDS coverage of all three phages was 95%, of which only approximately 40% of the CDS were annotated with known functions in all three phages. The rest of the genes were classified as having hypothetical functions. All three phage genomes lacked genes encoding known toxins, antimicrobial-resistance genes (ARGs), virulent factors (VFs) of bacterial origin, and phage lysogenic markers such as integrases, recombinases, repressor/anti-repressor proteins, and excisionases. Further, no tRNA, tmRNA, and CRISPRs were found (Table 1). Moreover, the in silico PhageAI tool (https://phage.ai/) categorized all three phages as strictly virulent (lytic) with high confidence (~ 96%). Hence, we consider all three phages to be excellent candidates for biocontrol of *E. coli* infections.

**Fig. 4:**
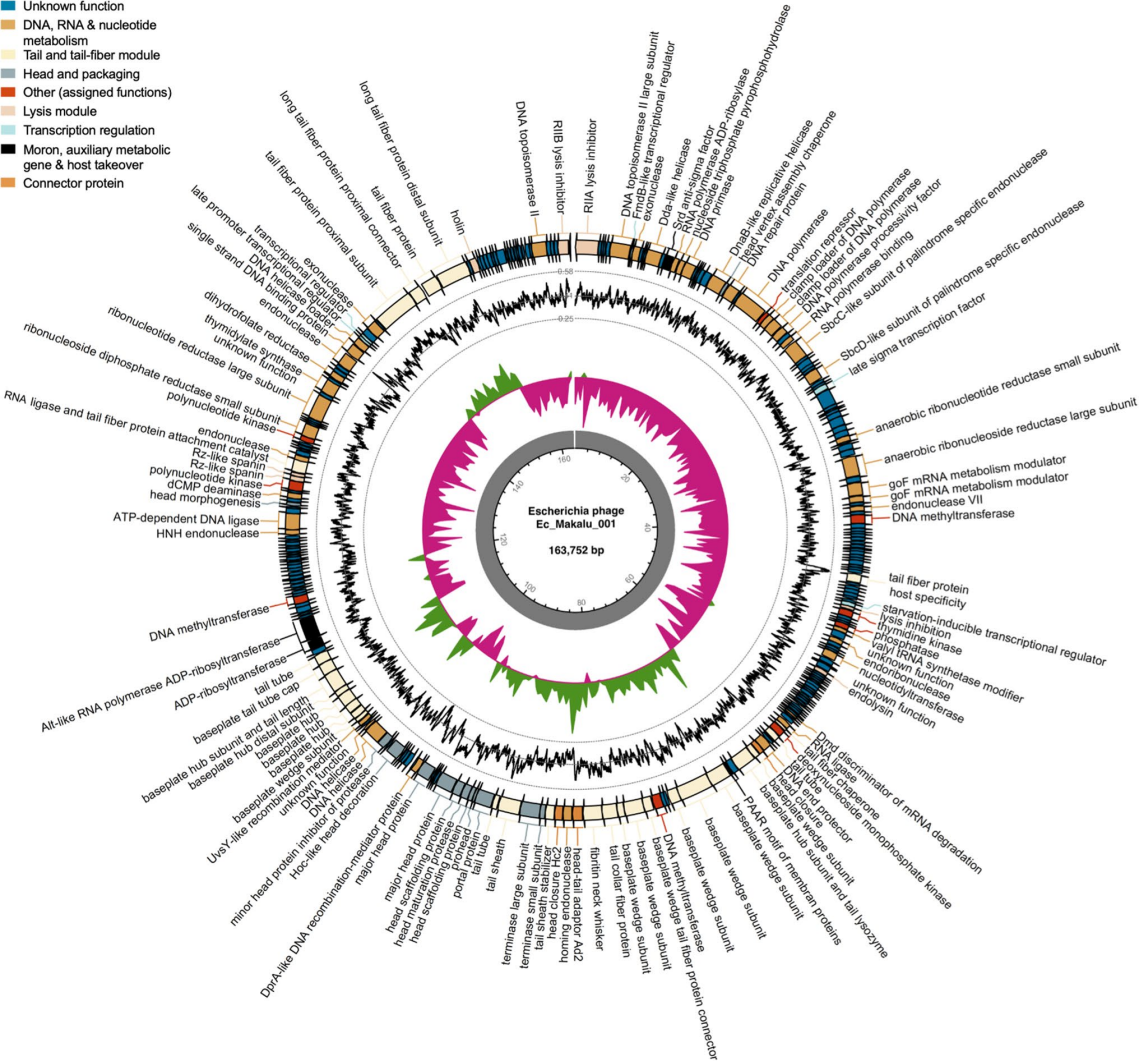
The whole-genome map of Escherichia phage Ec_Makalu_001. The outer circle with arrow-headed bands represents the coding DNA sequences (CDS) of Escherichia phage Ec_Makalu_001 color-coded according to the functional category of the predicted gene in the direction of the transcription. The innermost ring represents the genome backbone, and next is the GC skew (green/pink, window = 500 bp) followed by GC content (black, window = 10 bp). The labels show the predicted functions of the functional CDSs, color coded by PHROGs category

**Fig. 5:**
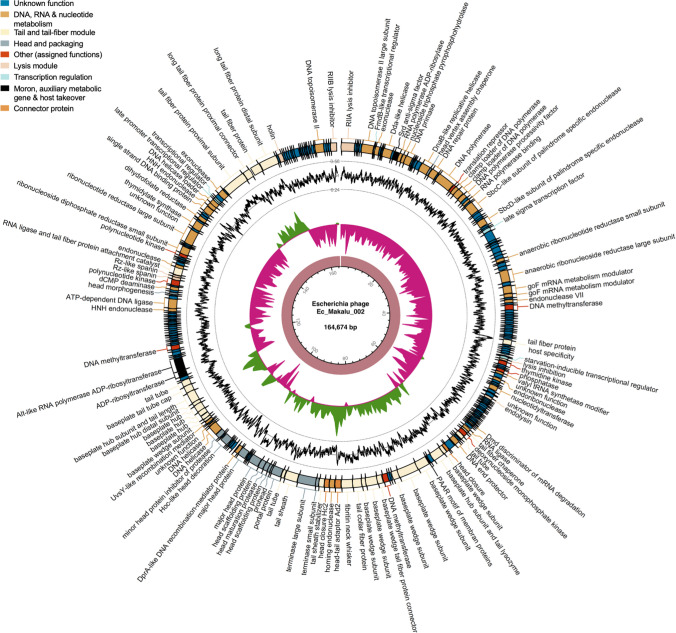
The whole-genome map of Escherichia phage Ec_Makalu_002. The outer circle with arrow-headed bands represents the coding DNA sequences (CDS) of Escherichia phage Ec_Makalu_002 color-coded according to the functional category of the predicted gene in the direction of the transcription. The innermost ring represents the genome backbone, and next is the GC skew (green/pink, window = 500 bp) followed by GC content (black, window = 10 bp). The labels show the predicted functions of the functional CDSs, color coded by PHROGs category

**Fig. 6:**
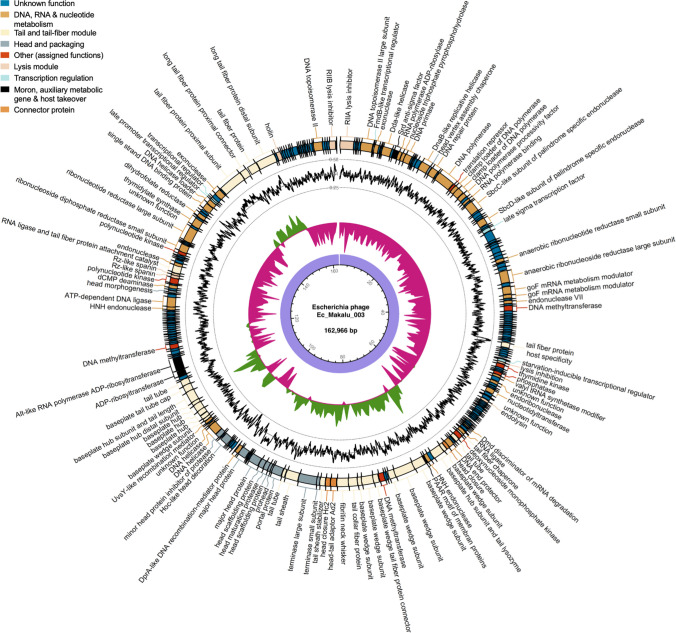
The whole-genome map of Escherichia phage Ec_Makalu_003. The outer circle with arrow-headed bands represents the coding DNA sequences (CDS) of Escherichia phage Ec_Makalu_003 color-coded according to the functional category of the predicted gene in the direction of the transcription. The innermost ring represents the genome backbone, and next is the GC skew (green/pink, window = 500 bp) followed by GC content (black, window = 10 bp). The labels show the predicted functions of the functional CDSs, color coded by PHROGs category

**Fig. 7:**
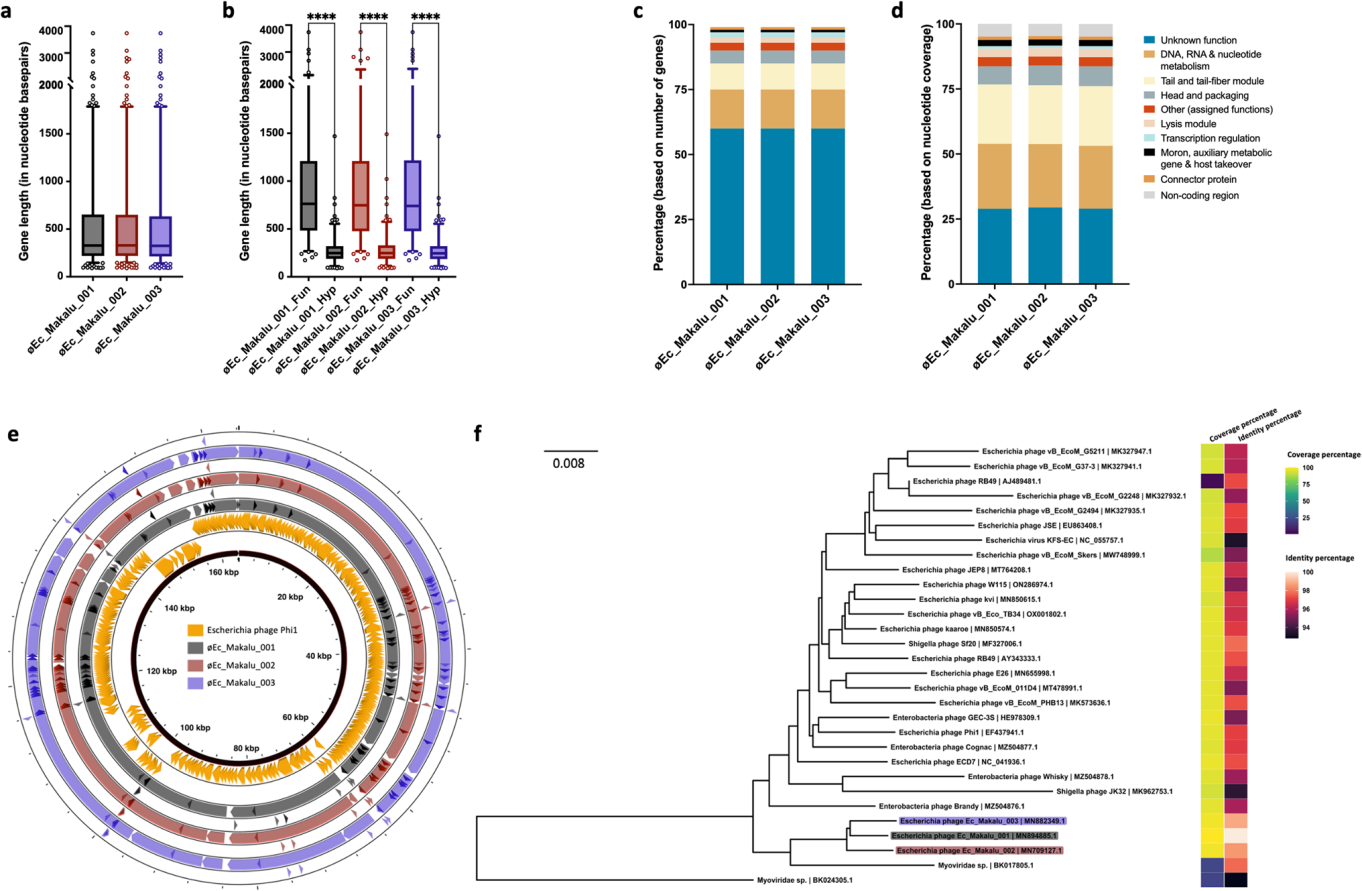
Comparative genomic features of isolated phages. **a** Average gene length of isolated phages. **b** Average gene lengths of functional and hypothetical genes. **c** Percentages of CDS categories according to gene numbers. **d** Percentages of CDS categories according to their genome coverage. **e** Whole genome comparison of isolated phages with the reference genome (Escherichia phage Phi1). The color in the similarity profile corresponds to the conserved region in that particular genome region while the blank regions (white) represent fragments that are not aligned or conserved in a particular genome sequence. **f** Phylogenetic relatedness of isolated phages with other similar phages in NCBI database (*N* = 27). The phylogenetic tree was constructed using the fast-minimum evolution method

Based on the specific locations of the predicted genes, associated functions and overall organization of the genomes, typically comprising DNA packaging mechanism, GC content, DNA replication-transcription, and structural genes, all three phages shared a modular structure with many phages within the *Straboviridae* family (traditionally *Myoviridae* morphological family which is now abolished) (Turner et al. 2023), more specifically, with phages belonging to the genus of T4-like viruses with high similarity to the RB49 group virus (Petrov et al. 2010). Genes encoding putative holin, endolysin, and spanin complex (i-spanin/o-spanin) involved in the host bacteria lysis were scattered throughout the genomes of all three phages (Figs. 4, 5, 6). The average gene length in all three phages was highly similar and ranged between 546 and 551 base pairs (Fig. 7a). Compared to functional genes, the average lengths of hypothetical genes in all three phages were significantly lower (Fig. 7b, p < 0.001, *t*-test) suggesting them to be either non-functional false positives or part of the small-phage gene family noted recently by Fremin et al. (2022). Upon further analysis, we noticed that although almost 60% of the total genes were hypothetical (unknown function) in all three phages, the total nucleotide (genome) coverage by those hypothetical genes was lower (~ 29%) (Fig. 7c–d) indicating the hypothetical CDS to account for far lesser genome coverage than functional genes. Most of the phage genome encoded genes for DNA, RNA metabolism (~ 24%), and structural proteins like tail and tail fiber (~ 23%) followed by head and packaging (~ 7%) (Fig. 7d).

### Comparative genome analysis and phylogeny

Further, comparative genome analysis of all three *Escherichia* phages revealed significant homology within each other and a high degree of nucleotide similarity (98%) (Fig. 7e and Table S1). Also, our phages were phylogenetically close to Enterobacteria phage Phi1 (query coverage = 96%, percent identity = 97.07%) (GenBank: EF437941). Further BLAST comparison of øEc_Makalu_001, øEc_Makalu_002, and øEc_Makalu_003 with Enterobacteria phage Phi1 revealed that the essential structural genes, DNA replication, repair and recombination module, phage DNA packaging genes, and genes of auxiliary metabolism show a high level of nucleotide identity despite their different geographical origin (Fig. 7e). The genome of øEc_Makalu_001 shared 97% nucleotide similarity with both øEc_Makalu_002 and øEc_Makalu_003 and 93.19% with Enterobacteria phage Phi1. Similarly, øEc_Makalu_002 shared 96.1% identity with øEc_Makalu_003 and 91.2% with Enterobacteria phage Phi1. Ninety-one percent nucleotide identity was observed between øEc_Makalu_003 and Enterobacteria phage Phi1. Multiple genome alignment and progressive MAUVE comparison of isolated phages with each other revealed similar synteny and high homologous regions at protein levels, typically genes encoding DNA packaging/replication/transcription regulation, structural proteins like head, tail fiber, and lytic cassettes (Figs. 4–6 and Fig. S1).

MAUVE comparison of all 3 phages with the reference Enterobacteria phage Phi1 revealed that all these genomes are highly co-linear and related except for the deletion of seven genes of the Enterobacteria phage Phi1 (ORF84.1, ORF110, ORF111, ORF130.1, ORF131.1, ORF257, and ORF263.1) in the genomes of øEc_Makalu_001, øEc_Makalu_002, and øEc_Makalu_003 phages. Likewise, hypothetical protein-coding genes, i.e., ORF 232, ORF237 of øEc_Makalu_001 homolog to ORF233, ORF238 of øEc_Makalu_002 and ORF233, ORF239 are absent in the genome of the Enterobacteria phage Phi1. Further, ORF238 of øEc_Makalu_001 and its homolog ORF240 of Enterobacteria phage Phi1 encoding a hypothetical protein is deleted in the genomes of øEc_Makalu_002 and øEc_Makalu_003. Interestingly, øEc_Makalu_002 genome possesses two alleles of HNH-type homing endonucleases (ORF131 and ORF240) which are absent in phage øEc_Makalu_001 and Enterobacteria phage Phi1, but only one HNH-type endonuclease (ORF136) is conserved in øEc_Makalu_003.

Further, a comparative phylogenetic analysis of our phage genomes in the NCBI nucleotide database (nBLAST) revealed that the phage genomes had substantial homology with 24 other phage genomes available in the NCBI database (coverage = 88–97%, identity = 93–98%, total identity = 84–97%) (Table S1) and distantly related to 3 Myoviridae sp. (partial genome, total identity = 19–21%) (Fig. 7f). All three phages (øEc_Makalu_001, øEc_Makalu_002, and øEc_Makalu_003), which were isolated from Kathmandu, Nepal, fall within the same family, genus, and species level clusters (F1, G1, and S6) (Fig. S3).

## Discussion

The emergence of multi-drug-resistant organisms (MDROs), including *E. coli*, is becoming a public health threat. As such, there is a renewed interest in developing alternative strategies to combat the global burden of antibiotic resistance. Among many alternatives, phages and/or their components (lysin) are considered one of the potential and feasible alternatives, as phages are ubiquitous (Romero-Calle et al. 2019). Well-characterized phages are required for therapeutic use to ensure the genomic safety of the therapeutic phage as some lysogenic phages encode toxin-producing genes like Shiga toxin (*stx*) by Stx phages*,* cholera toxin (*ctx*) by CTX phages. Recently, AMR genes and other VFs have also been reported in transducing phages (prophages), which modulate bacterial lifestyle, virulence, and pathogenesis (Philipson et al. 2018; Nepal et al. 2022b, 2023). In the environment, the presence of phage depends upon the availability of their host bacterium. River water, sewage, soil, poultry manure, stagnant water, and sea water are rich sources of phages as they usually contain bacteria (Mulani et al. 2019). We primarily collected sewage from Kathmandu, Nepal, as a phage source because it is extensively contaminated with untreated waste from industries, hospitals, and households, creating a favorable niche for the dynamic co-evolution of phage and bacteria. This may equip phages with novel genetic backgrounds. As a result, a novel phage that is more effective against the pathogens in such a niche may evolve. Earlier, we isolated and studied the efficacy of virulent phages infecting *Klebsiella pneumoniae* and *Pseudomonas aeruginosa* (Dhungana et al. 2021a, 2021b; Maharjan et al. 2022). In this study, we isolated three phages, namely øEc_Makalu_001, øEc_Makalu_002, and øEc_Makalu_003, all belonging to *Straboviridae* family that could effectively lyse 28–34% of uropathogenic *E. coli* clinical isolates. All three phages produced clear plaques on their host lawn and were devoid of any ARGs, VFs, toxin-encoding genes, and lysogeny modules in their genomes. Thus, all three phages were considered strictly lytic, having potential for therapeutic applications.

Burst size (average number of phage particles released per infected bacterium) is an important parameter when selecting phages for efficient and effective phage therapy (Khan Mirzaei and Nilsson 2015). Our results revealed that the latent periods of the 3 phages were between 15 and 20 min, with a burst size of up to 127 phages/bacterium. The latent period, the time it takes for phage to induce the lysis of the host cell, depends on multiple factors like host physiology, and phage lytic protein complex like holin, endolysin, and spanin (Rajaure et al. 2015; Abedon et al. 2001). Phage having a short latent period (~ 20 min) and high burst size is better suited to eliminate infecting bacteria as well as overcome the risk of development of phage-resistant mutants. Here, all phages øEc_Makalu_001, øEc_Makalu_002, and øEc_Makalu_003 showed promising lytic properties with short latent periods and with high burst sizes clearly showing potential for therapeutic applications.

The stability of phages in different physiochemical conditions is another important factor for storage and applications (Jonczyk et al. 2011). All phages used in this study were stable at 25 °C and 37 °C and between pH 6–9 without significant loss of phage titers. These results corroborate the findings of a great deal of previous work. Tailed phages (T4, T5, and T7) are known to be robust, survive in adverse conditions for several years, and thus are preferred in therapeutics (Ackermann et al. 2004). Previous studies have shown that tailed Myophage remained stable at 4–37 °C, decreased infectivity at 60–70 °C within 15 min, and was completely inactivated at 50 °C for 60 min or 80 °C for 15 min (Zhao et al. 2019). Similarly, T4 phage showed optimum stability at pH 6.0–7.4 and decreased its titer at above pH 9.2 and below pH 4.0 (Jonczyk et al. 2011). Our results agree with these findings and thus we conclude that our phages can be stored at optimum pH and temperatures without significant loss of its viability.

Further, the host range spectrum of a phage is critical in the selection of therapeutic phage and usually phages with a broad host range are preferred for therapeutic applications. Though phages are conventionally regarded as extremely specific to their host which limits their application in phage therapy, occasionally broad host range phages have been reported. Recent findings have shown that some phages possess the ability to infect a wide range of bacterial strains, including intergeneric lysis (Malki et al. 2015; Fernandez et al. 2019; Göller et al. 2021). Thus, the host range of isolated *Escherichia* phages was screened against isolates representing 3 different bacterial genera (*Escherichia*, *Klebsiella*, and *Pseudomonas*) followed by EoP to determine the relative EoP. Based on spot assay and EoP, all three phages showed broad host killing within its host genus *Escherichia* so were considered Wide Host Range (WHR) phages. Interestingly, øEc_Makalu_002 and øEc_Makalu_003 also showed intergeneric lysis producing faint lysis zone on the lawns of *K. pneumoniae* isolates (EoP = 0.0–0.2). This suggested property of polyvalent phages displaying remarkable host diversity by Myophages. Similar polyvalent WHR phages have also been reported in the past. Bielke et al. (2007) showed that lytic phage isolated using *Salmonella enteritidis* was also successfully amplified in *Escherichia*, *Klebsiella*, and other strains of *Salmonella*. Similarly, Sui et al. (2021) reported that polyvalent phage isolated using *Escherichia coli* could also infect *Salmonella enteritidis* and Jensen et al. (1998) reported phages capable of intergeneric replication in *Sphaerotilus natans*, *E. coli*, and *P. aeruginosa* that could cross-infect each other. Similarly, Greene and Goldberg (1985) were able to isolate bacteriophages capable of lysing more than one species of *Streptomycetes*. Further, a polyvalent *Escherichia coli* phage ECD7 (RB49 group of viruses) having WHR (infecting enterotoxigenic *E. coli*, enteroaggregative *E. coli*, enterohemorrhagic *E. coli*, *Shigella flexneri*, and *Shigella sonnei*) has been used as a phage-based probiotic dietary supplement to treat Traveler’s diarrhea (Aleshkin et al. 2015). In this study, we also report WHR phages able to infect distinct host genera. Based on this, we postulate that phages can interact with multiple host genera in nature and evolve over time, showing different phenotypic plasticity suggesting complex co-evolutionary relationships between bacteria and phages. In phage therapy, a phage that can infect multiple genera of bacteria effectively is equivalent to broad-spectrum antibiotics (Ross et al. 2016) and is an extremely desirable property because bacteriophages would not have to be isolated for individual isolates. This evidence suggests that WHR phages exist in nature, and a small library of such phages could potentially treat a wide range of infections. Additionally, the amplification of bacteriophages in a nonpathogenic alternative host eliminates the possibility of incorporating detrimental accessory genes during phage amplification. The low EoP on *K. pneumoniae* isolates might be due to the release of bacteriocin or breakdown of cellular energetics leading to abortive infection (Kutter 2009). Further analysis is required to characterize those phages and their intergenus infective capacity more in depth.

The long tail fiber of the phage initially recognizes the receptors present on the host cell surface and facilitates the initial binding, which determines host specificity (Hyman and van Raaij 2018). Comparative sequence analysis of tail fiber protein at the amino acid level using pairwise sequence alignment revealed that øEc_Makalu_002 and øEc_Makalu_003 shares 80.1% sequence similarity (Fig. S2). Both phages have highly conserved identical amino acid sequences (100% identical) in N-terminus (up to 371 residues) and low identity in the C-terminus region. N-terminal residues of the tail fiber protein are responsible for attachment towards the baseplate, so this region may be highly consistent while C-terminal region recognizes the host receptors protein where receptor-binding domain is located. Significant differences in host range and other biological properties could be observed with subtle changes in the sequence in tail fiber proteins (Yosef et al., 2017). The putative long-tail fiber tip protein of each phage is comparatively different from each other at a nucleotide level and may make different host range. It is further hypothesized that phages capable of intergeneric lysis may use receptors, intermediary functions, or both common to a wide range of bacteria to achieve a wide host range.

Computational analysis of the genomes revealed that all three phages were virulent and free from known toxins, ARGs and VFs of bacterial origin. Further, øEc_Makalu_001, øEc_Makalu_002, and øEc_Makalu_003 genomes were highly homologous with the genomes of known members of T4-like viruses (representatives of the RB49 group, genus *Krischvirus*) (Walker et al. 2019). The RB49 group of viruses are pseudo-T-evens phages primarily infecting Enterobacteria and environmentally important as they are found ubiquitously (Monod et al. 1997) and till date, 19 phage genomes having more than 96% sequence identity with Enterobacteria phage RB49 have been deposited to NCBI GenBank. The genome size of all these phages ranged from 162,966 bp to 176,009 bp with a very small window of G + C content (40.32 to 40.68%) and were isolated using *Escherichia coli* as a primary host except two Shigella phages JK32 and Sf20. The genome size and G + C content of all 3 phages were also highly consistent with other phage genomes of RB49 group and shared > 96% ORF homology with each other. Interestingly, despite the phylogenetic relationship of the phages with each other and with other phages from RB49 group, they were isolated using different hosts and on different continents. A high degree of sequence similarity and identity may probably be due to the horizontal exchange of genes from a shared pool among the ancestors of contemporary phages during co-evolution (Hendrix et al. 1999). Thus, we assume that øEc_Makalu_001, øEc_Makalu_002, and øEc_Makalu_003 and their relatives may have co-evolved with their host through extensive gain, loss, and exchange of their genes under different selection pressures and diverged from a common ancestor. Homing endonucleases play an important role in homologous recombination between phages during co-infection with the same host. The variable number of HNH-type endonucleases found in the 3 phage genomes possibly have a role in the genome evolution of the phages. Further experiments are needed to unravel the exact role of these endonucleases and the significance in diversification and evolution of the phage genome over time.

## Conclusions

In summary, all three isolated phages exhibited excellent anti-*E. coli* activities, high thermal and acid tolerance, fulfilled all the available essential genomic safety checkpoints for T4-like phage and qualified as a potential therapeutic phage candidate. All three phages shared conserved modular genome architecture similar to T4-like phages and shared a common ancestral sequence. Despite high sequence homology with each other, additional unique ORFs of unknown ortholog were identified within their genomes, possibly acquired by the mechanism of lateral gene transfer during evolution, as it is established that T4-like phages do not undergo lysogeny. This library would be valuable for selecting appropriate phage for future phage therapy, used alone or in phage cocktails, and future research work. Thus, regular isolation of phages from diverse ecology and geographical areas is necessary to understand their sequence diversity in phage databases.

## Supplementary Information

Below is the link to the electronic supplementary material.

Supplementary file1 (PDF 912 KB)

## Data Availability

The original results of the study are included in the article. Additional data are included as supplementary data. Further inquiries can be directed to the corresponding author(s).

## Declarations

### Limitations

We could not elucidate the mechanism underlying the ability of øEc_Makalu_002 and øEc_Makalu_003 to lyse *K. pneumoniae* (NK and K23) as this was beyond the scope of this project. Further research is required to elucidate the mechanism for inter-genus host lysis capability which may help us engineer synthetic phages with the ability to lyse wider hosts.
