# Supplementary material for: Preclinical characterization and in silico safety assessment of three virulent bacteriophages targeting carbapenem-resistant uropathogenic Escherichia coli
Source: Int Microbiol. 2024 Mar 22;27(6):1747–63. doi: 10.1007/s10123-024-00508-8 (PMC11611945; doi:10.1007/s10123-024-00508-8)
Supplement: Supplementary file 1 — Supplementary file1 (PDF 912 KB) [file 10123_2024_508_MOESM1_ESM.pdf]

SUPPLEMENTARY MATERIALS

Characterization and preclinical *in silico* safety assessment of three virulent bacteriophages targeting carbapenem-resistant uropathogenic *Escherichia coli*

Gunaraj Dhungana [1,2*], Roshan Nepal [3,4,5*], Ghais Houtak [3,4], George Bouras [3,4], Sarah Vreugde [3,4], Rajani Malla [1]

[1] Central Department of Biotechnology, Institute of Science and Technology, Tribhuvan University, Kirtipur, Nepal

[2] Nepal Health Research Council, Government of Nepal, Kathmandu, Nepal

[3] Adelaide Medical School, Faculty of Health and Medical Sciences, The University of Adelaide, Adelaide, SA, Australia

[4] The Department of Surgery-Otolaryngology Head and Neck Surgery, The Basil Hetzel Institute for Translational Health Research, Central Adelaide Local Health Network, South Australia, Australia.

[5] The Commonwealth Scientific and Industrial Research Organization (CSIRO), Agriculture and Food, Hobart, Tasmania, Australia

Supplementary figure(s)

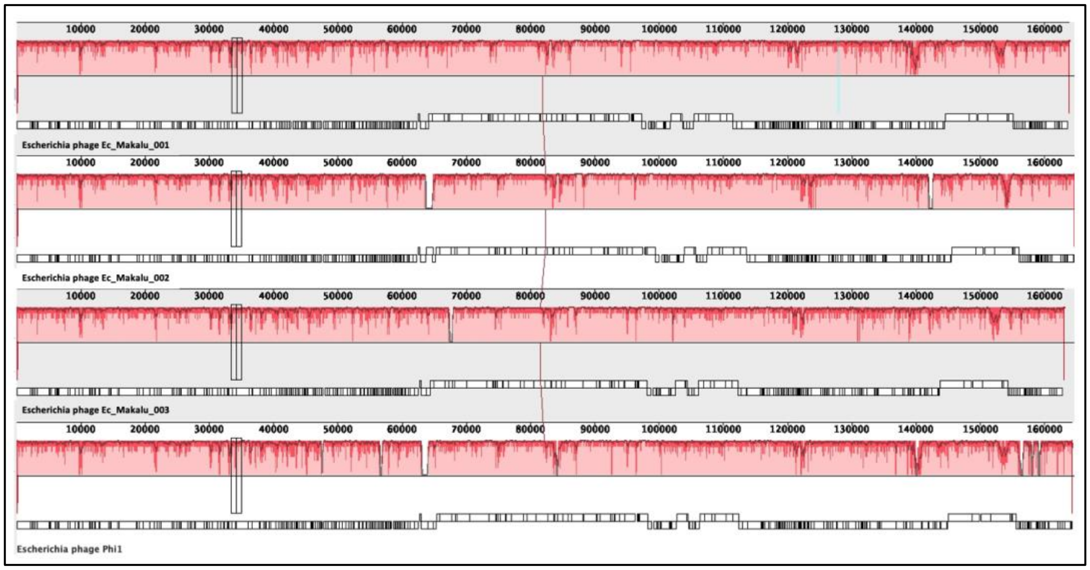

Fig. S1 | MAUVE alignment of three Escherichia phages (øEc_Makalu_001, øEc_Makalu_002 and øEc_Makalu_003) compared with the most closely related Escherichia phage Phi1.

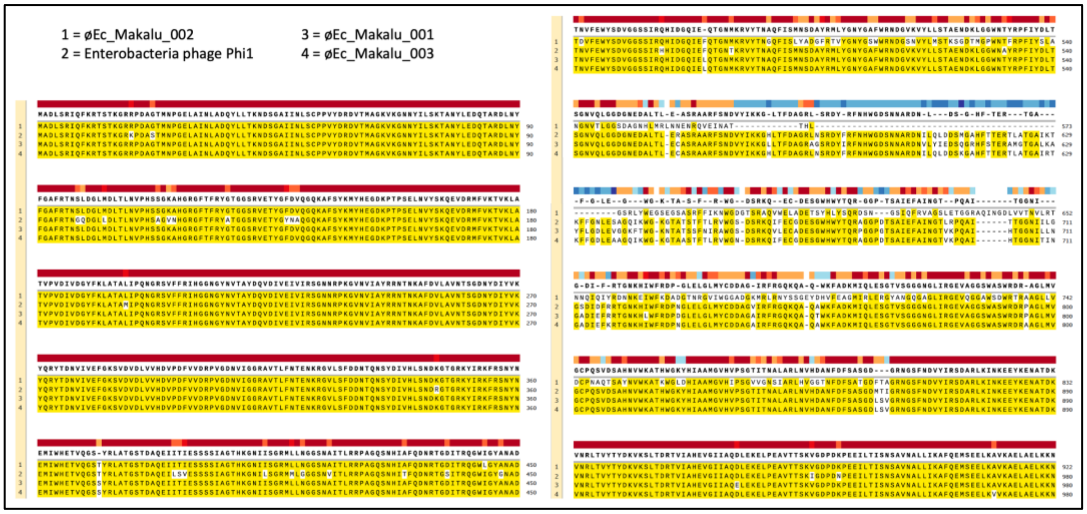

Fig. S2 | Consensus alignment of long tail fiber protein. Amino acids that match the reference are marked with yellow highlighting. Consensus threshold = 50%.

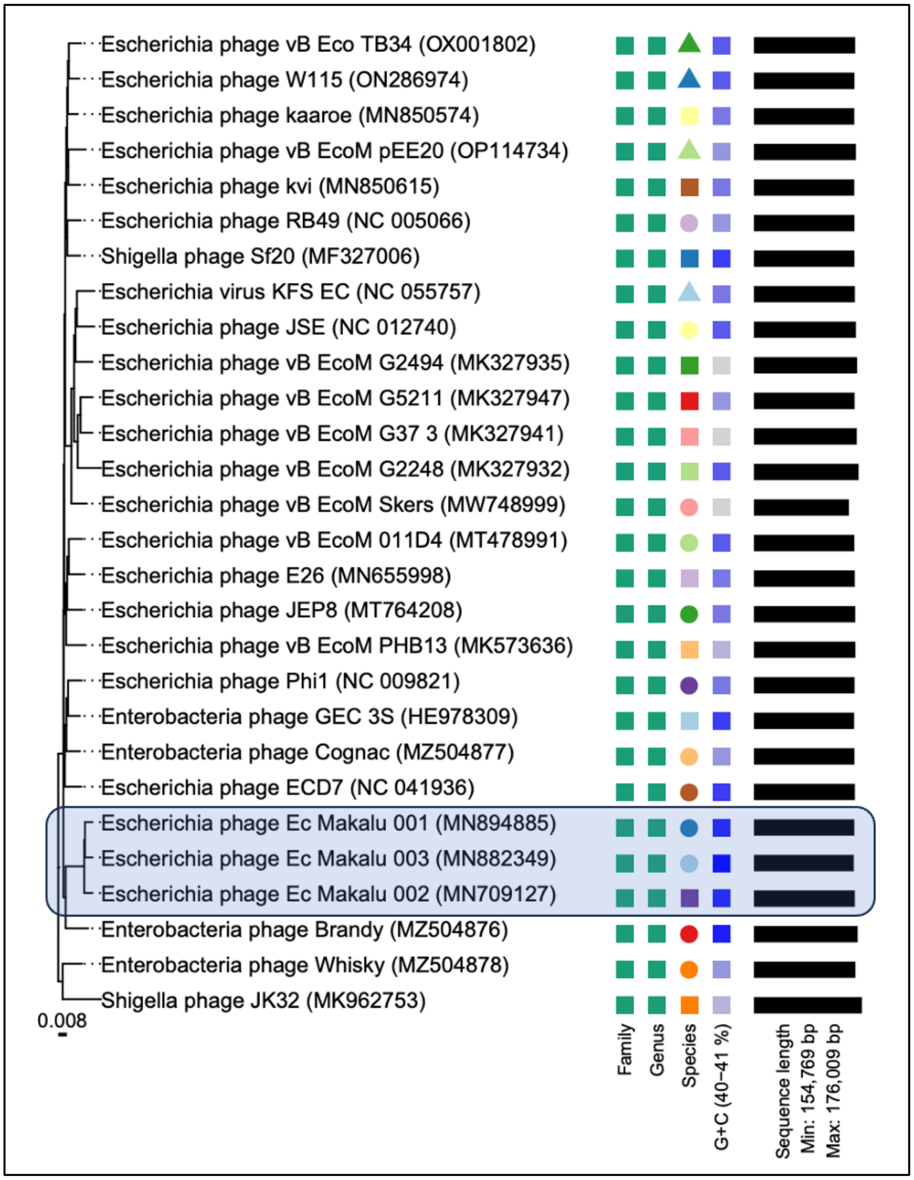

Fig S3 | The Genome-BLAST Distance Phylogeny (GBDP) tree of phages øEc_Makalu_001, øEc_Makalu_002, and øEc_Makalu_003 inferred using formula D4 and yielding average support of 6 %. The analysis reveals that all three phages belong to the same family and genus level and share maximum sequence similarity with other phages in the NCBI database, including Shigella phages.

Supplementary table(s)

Table S1 | Sequences producing significant alignments with reference genome of Escherichia phage Ec_Makalu_001

SN	Scientific Name	Accession	Max Score	Total Score	Query Coverage	Per. identity	Total Per. identity	Acc. Length
1	Escherichia phage Ec_Makalu_001	MN894885.1	3.02E+05	3.05E+05	100%	100	100.00	163752
2	Escherichia phage Ec_Makalu_002	MN709127.1	1.12E+05	2.87E+05	98%	98.49	96.52	164674
3	Escherichia phage Ec_Makalu_003	MN882349.1	77484	2.89E+05	98%	98.79	96.81	162966
4	Escherichia phage Phi1	EF437941.1	62462	2.59E+05	96%	97.07	93.19	164270
5	Escherichia phage kvi	MN850615.1	58362	2.55E+05	94%	96.74	90.94	163673
6	Escherichia phage JEP8	MT764208.1	57899	2.59E+05	96%	96.51	92.65	165295
7	Escherichia phage vB_EcoM_PHB13	MK573636.1	55118	2.56E+05	96%	97.31	93.42	165641
8	Enterobacteria phage GEC-3S	HE978309.1	51849	2.61E+05	97%	95.04	92.19	163424
9	Escherichia phage ECD7	NC_041936.1	49807	2.59E+05	97%	97.29	94.37	164706
10	Escherichia virus KFS-EC	NC_055757.1	49748	2.47E+05	94%	93.29	87.69	164715
11	Escherichia phage vB_EcoM_011D4	MT478991.1	49543	2.59E+05	96%	95.1	91.30	163764
12	Escherichia phage RB49	AY343333.1	43674	2.60E+05	96%	97.33	93.44	164018
13	Escherichia phage kaaroe	MN850574.1	43120	2.60E+05	96%	96.94	93.06	163719
14	Escherichia phage E26	MN655998.1	42911	2.60E+05	96%	96.43	92.57	164572
15	Escherichia phage vB_EcoM_G2248	MK327932.1	42475	2.38E+05	93%	95.48	88.80	170678
16	Escherichia phage vB_EcoM_Skers	MW748999.1	41539	2.33E+05	88%	95.08	83.67	154769
17	Enterobacteria phage Brandy	MZ504876.1	40470	2.57E+05	96%	95.83	92.00	169232
18	Escherichia phage W115	ON286974.1	39548	2.61E+05	96%	95.18	91.37	163997
19	Shigella phage JK32	MK962753.1	38428	2.29E+05	93%	93.65	87.09	176009
20	Escherichia phage vB_EcoM_G37-3	MK327941.1	37096	2.44E+05	94%	96	90.24	167832
21	Enterobacteria phage Whisky	MZ504878.1	34915	2.46E+05	95%	95.62	90.84	165615
22	Shigella phage Sf20	MF327006.1	33787	2.60E+05	96%	97.75	93.84	163982
23	Escherichia phage vB_Eco_TB34	OX001802.1	33606	2.62E+05	96%	96.56	92.70	165220
24	Enterobacteria phage Cognac	MZ504877.1	31490	2.53E+05	95%	96.94	92.09	164031
25	Escherichia phage vB_EcoM_G5211	MK327947.1	31242	2.40E+05	93%	96.21	89.48	164278
26	Escherichia phage vB_EcoM_G2494	MK327935.1	28801	2.45E+05	94%	97.15	91.32	168327
27	Escherichia phage JSE	EU863408.1	27253	2.45E+05	95%	96.97	92.12	166418
28	Myoviridae sp.	BK017805.1	24912	59131	22%	97.7	21.49	40781
29	Myoviridae sp.	BK016552.1	16460	47240	24%	89.23	21.42	43086
30	Myoviridae sp.	BK016985.1	11300	11300	5%	90.13	4.51	12469
31	Myoviridae sp.	BK024305.1	7638	46483	21%	92.82	19.49	38377
32	Escherichia phage RB49	AF203974.1	3251	3251	1%	98.79	0.99	1824
33	Escherichia phage RB49	AJ489481.1	3020	3020	1%	97.25	0.97	1783
34	Escherichia phage RB49	AB084150.1	2900	2900	1%	95.73	0.96	1803
35	Escherichia coli	CP071263.1	2765	3398	1%	85.4	0.85	5241795
36	Escherichia phage RB49	AJ550428.1	2488	2488	0%	96.78	0.00	1491
37	Escherichia coli	CP057368.1	2183	3209	2%	81.65	1.63	4743602
38	Escherichia coli	CP028733.1	1978	2617	1%	80.31	0.80	4816573
39	Escherichia coli	CP063720.1	1930	2541	1%	79.99	0.80	4771390
40	Escherichia coli	CP057861.1	1925	2585	1%	79.95	0.80	4899428
41	Escherichia coli	CP057166.1	1842	2901	2%	79.41	1.59	4935063
42	Escherichia coli	CP091038.1	1646	3133	2%	84.75	1.70	4911746
43	Enterobacteria phage MV BS	DQ485346.1	1290	1290	0%	95.76	0.00	799
44	Escherichia coli	CP024131.1	1229	2819	1%	83.54	0.84	5084741
45	Escherichia fergusonii	CP057093.1	1201	2777	1%	82.99	0.83	4730551
46	Myoviridae sp.	BK030387.1	1184	2199	1%	82.03	0.82	36350
47	Escherichia fergusonii	CP079891.1	1109	2645	2%	83.67	1.67	4934492
48	Shigella dysenteriae	CP055055.1	1086	1086	0%	83.35	0.00	5075418
49	Shigella dysenteriae	CP055052.1	1086	1086	0%	83.35	0.00	5075418
50	Myoviridae sp.	BK043428.1	1081	1081	0%	83.25	0.00	36352
